# Conference survival guide—the dos and don’ts

**DOI:** 10.1038/cddiscovery.2016.88

**Published:** 2016-11-21

**Authors:** Tatiana P Soares da Costa

**Affiliations:** 1 Department of Biochemistry and Genetics, La Trobe Institute for Molecular Science, La Trobe University, Melbourne, Victoria 3086, Australia

One of the perks of doing research is being able to attend conferences, especially if they are held in idyllic locations, as they often are. As a postgraduate student or early career researcher, you are often encouraged to attend conferences to promote your research, receive feedback on your work, share ideas with peers and practice your science communication skills.^1,2^ It may seem daunting having to present in front of a group of experts in your field and you may concoct excuses why you should not attend one – but frankly that's what they are, just excuses. How many chances do you really get to discuss your work with people who are just as passionate as you and have the time to do it? One of the strange things is that often you find out about work from your own institute and talk to people whom you already know well at conferences and finally get to hear about their fantastic work. But this is what happens when you are no longer running around balancing family, work, deadlines and meetings, and just have a moment to think. Here are a few tips on how to get the most from your conference attendance:

## Choosing the right conference

### Do

- Attend a conference where you have the opportunity to present your work, either as a poster or an oral presentation.^3^ That way you feel like an active participant rather than just a spectator and it is an achievement to put on your resume. However, even if you do not present, you can always ask questions either in the session or during a coffee break. There are many ways to get involved.
- Choose a conference that is more focused on your area of research. This might make networking less daunting. Nevertheless, attending a conference on a slightly unrelated topic can stimulate you with fresh ideas and approaches.
- Ask advice from your supervisor about the best meeting to attend. After all, it is always best to seek their permission first and they often help financially (see below). They might even be able to put in a good word for you with the organisers.
- Attend small local conferences. They may not be as glamorous but they are a great place to dip your toe into the water. Conferences in more isolated locations, where the meals are provided by the conference organisers and eaten together, are a great way to really get to know the attendees. These small conferences are a great option for first year PhD students and often less expensive.

### Don’t

- Choose a conference that nobody has ever heard about before. There is a worryingly increasing number of fake conferences and these are just a waste of time.
- Choose a conference based on location or because it is a convenient time of the year to go. If you want a holiday, have a holiday but a conference is, even when held in a beautiful location, just another way of working and you should treat it like a job.

## Apply for funding

Conferences can be expensive so it is always a good idea to look for funding support.

### Do

- Apply for departmental or university travel fellowships. Keep an eye on the application deadlines so you do not miss the opportunity to apply.
- Become a member of a professional society. Many societies have fellowships to assist recipients to attend overseas conferences. For example, the American Association of Immunologists recently advertised a million dollars of travel support. Bear in mind that for a lot of the awards you need to be a member of the society for a period of time before being eligible to apply.
- Look for travel fellowships awarded by philanthropic organisations.

### Don’t

- Expect your supervisor to cover all the costs. Your supervisor may have some funding set aside to allow members of the lab to attend conferences but this should not be an expectation. Having said that, most supervisors are aware of the benefits of attending a scientific conference and will support you attending a good quality conference.

## Registration process

### Do

- Register before the early bird registration closes (typically ~3 months before the conference kicks off). Booking in advance not only keeps your registration price lower but will also keep your travel and accommodation costs lower.
- Have your abstract ready far enough in advance to allow your supervisor and collaborators to review it well before the deadline.

### Don’t

- Miss the early bird deadline. A lot of the time, the early bird deadline coincides with the oral presentation deadline and you do not want to miss the opportunity to be considered for an oral presentation.
- Write your abstract at the last minute. Being considered for a presentation usually comes down to a well written abstract.^4^

## Conference preparation

### Do

- Keep up with the literature, so you have an idea what to expect.
- Read the conference abstracts and select any talks or poster presentations you want to attend. Typically, the final schedule for a conference will be posted about a month in advance. Pick out presentations that sound interesting and create a schedule for each day. A lot of conferences nowadays have apps that you can download to keep a track of your schedule.^5^ Read papers in advance from speakers whom you are especially interested in, to help you retain and understand better when they do present.
- Learning more about the speakers' research will also help you if you have the opportunity to speak to any of them. Imagine how impressive you will sound if you can discuss their own work with them rather than the weather or the food.
- Prepare, prepare, freak out, prepare for your presentation. It is always a good idea to have a rehearsal in front of your lab group, your granny, your dog and have them think of possible questions you may get (ok, the dog cannot ask questions). If you are presenting a poster, it would be useful to practice how you may strike up a conversation with someone who comes past about your research so you can receive invaluable one-on-one feedback.
- Bear in mind that people usually ask you questions because they are genuinely interested and want to know more about your work. You should not take it as an interrogation session or trap to catch you out on something.

### Don’t

- Plan a busy schedule. A conference programme may be overwhelming, particularly if you attend a large conference with plenary lectures, parallel sessions and multiple poster sessions. Try to find a balance between attending interesting presentations and leaving some free time in your schedule to process the information and network.
- Overstress about your presentation. Rehearse but do not obsess. When you become so stressed that you cannot stop thinking about it every day leading up to the conference, it is time to stop.

## Attending a session

Enthusiasm is usually at its highest at the start of the conference but that can quickly go away when you attend an uninteresting session or towards the end of a conference.

### Do

- Take a pen and notepad with you at all times.
- Plan to stay for all presentations in a session. Some presentations may be easier to understand than others but you never know what you may learn.
- Think of questions to ask. Even if you do not have the courage to ask them, it may help you stay alert. You may also have the opportunity to ask the questions later during a break.
- Attend the poster session. It is a great way to have one-to-one interaction with the presenter, so you cannot only provide feedback but also get ideas for your own research. Walking around a poster session will allow you to learn about recent discoveries and could help you find potential laboratories and institutions that you would like to work within the future.

### Don’t

- Fall sleep. If you are feeling tired before the start of a session, sit near an exit so you can quietly sneak out when tiredness gets the better of you.

## Network, network, network!

### Do

- Have informal discussions with people who attended your presentation. They may come up with useful feedback and it is a good opportunity to strengthen your connections.^6^
- Sell yourself. You may be finishing your PhD or looking for a new post-doc position. Meeting members of other labs may help you make a more informed decision about your next career move. Listen to what research other people are doing and think about what you would like to learn/do in your next position.^7^
- Have a chat to speakers, they may be your future employer.
- Networking should not only be done with more senior people but also with your peers, as they may eventually become your collaborators, reviewers, colleagues, employees or employers over your academic career.
- Get involved in social activities. Although it may be tempting to just hang out with people you already know, try to interact with new people. Chat to people in the conference room, during coffee breaks and meals. It is always a good idea to participate in activities organised by the conference, instead of disappearing into your room. You can also go to dinner or a drink with your new contacts because the best conversations usually happen in more relaxed environments. Even if you are shy by nature, try your best to meet and talk to as many people as possible, whether they be students, post-docs or professors. You will likely find that most people are quite friendly and willing to engage, share the same passion for science and have probably just heard the same talks.

### Don’t

- Spend all your free time getting free stuff from the industry booths. Free pens and USB sticks and so on, usually end up in the back of your wardrobe if they even make it back home with you.
- Burn yourself out. Although it may not seem like it, listening to endless talks and networking can be very tiring. Make the time to relax.

## Post conference

- When you return from your conference, before going back to everyday routine, spend some time going through the programme and have a look at your notes. Have a think about how you can put some of the ideas you had into action. It is also important to solidify the contacts you made by sending a follow-up email.
- Give a conference report to your lab.

- If you are lucky enough, write up a conference report for publication. Journals like Cell Death & Differentiation, Cell Death & Disease and Cell Death Discovery often commission 1–2 page conference reports. They are a great way to maintain your contacts and get an extra publication.^8–10^ What is not to like?

I hope these tips can make your next conference experience more enjoyable. Remember to just relax and have fun. You will be surrounded by people who are just excited about science as you are. Lastly, take the time to learn and discover a little bit about the place and country where you are staying. Mixing work with pleasure is one of the pros of being a research scientist after all!

## References

[bib1] Leslie JL. Nursing 2015; 45: 48–51. 10.1097/01.NURSE.0000458921.72726.17 25532985

[bib2] Smiljanic J et al. PloS One 2016; 11: e0148528. 26859404 10.1371/journal.pone.0148528 PMC4747514

[bib3] Woolston C. Nature 2016; 536: 115–117. 27494008 10.1038/nj7614-115a

[bib4] Varpio L et al. Med Teach 2016, In press.

[bib5] Kwok R. Nature 2013; 498: 395–397. 23789123 10.1038/nj7454-395a

[bib6] Knight M. J Environ Health 2011; 74: 4–5. 22187852

[bib7] Sohn E. Nature 2015; 526: 729–731. 26516640 10.1038/nj7575-729a

[bib8] Hildebrand JM et al. Cell Death Differ 2013; 20: 1593–1594. 24013723 10.1038/cdd.2013.121 PMC3792437

[bib9] Tentler et al. Cell Death Dis 2013; 4: e475. 23348589 10.1038/cddis.2012.209 PMC3563997

[bib10] von Karstedt S, Peltzer N. Cell Death Differ 2014; 21: 1343–1344. 24722211 10.1038/cdd.2014.43 PMC4085531

